# Repeatability and consistency of individual behaviour in juvenile and adult Eurasian harvest mice

**DOI:** 10.1007/s00114-017-1430-3

**Published:** 2017-02-24

**Authors:** Andrea C. Schuster, Teresa Carl, Katharina Foerster

**Affiliations:** Department of Comparative Zoology, Institute for Evolution and Ecology, University of Tübingen, Auf der Morgenstelle 28, 72076 Tübingen, Germany

**Keywords:** Animal personality, Repeatability, Consistency, Spatial cognition, Eurasian harvest mouse, *Micromys minutus*

## Abstract

Knowledge on animal personality has provided new insights into evolutionary biology and animal ecology, as behavioural types have been shown to affect fitness. Animal personality is characterized by repeatable and consistent between-individual behavioural differences throughout time and across different situations. Behavioural repeatability within life history stages and consistency between life history stages should be checked for the independence of sex and age, as recent data have shown that males and females in some species may differ in the repeatability of behavioural traits, as well as in their consistency. We measured the repeatability and consistency of three behavioural and one cognitive traits in juvenile and adult Eurasian harvest mice (*Micromys minutus*). We found that exploration, activity and boldness were repeatable in juveniles and adults. Spatial recognition measured in a Y Maze was only repeatable in adult mice. Exploration, activity and boldness were consistent before and after maturation, as well as before and after first sexual contact. Data on spatial recognition provided little evidence for consistency. Further, we found some evidence for a litter effect on behaviours by comparing different linear mixed models. We concluded that harvest mice express animal personality traits as behaviours were repeatable across sexes and consistent across life history stages. The tested cognitive trait showed low repeatability and was less consistent across life history stages. Given the rising interest in individual variation in cognitive performance, and in its relationship to animal personality, we suggest that it is important to gather more data on the repeatability and consistency of cognitive traits.

## Introduction

Over the last decades, the interest in individual variation in behaviour within animal species has increased rapidly. Consistent between-individual differences in behaviours, known as animal personality, coping styles or behavioural syndromes (Koolhaas et al. 1999; Sih et al. 2004; Réale et al. 2007), have been analysed in all main animal taxa to date. This is due to the relevance of animal personality as an important component of animal ecology and its effects on fitness (Thomas et al. 2016). In wild populations, animal personality could be linked to differences in reproductive success (Reale et al. 2000; Both et al. 2005), parental care (Budaev et al. 1999) and survival (Dingemanse et al. 2004; Boon et al. 2007), and even to different life history strategies (e.g. Vetter et al. 2016). Animal personality is specified as consistent between-individual behavioural differences throughout time and across situations (Réale et al. 2007). In many species, individuals behave repeatably when tested in the same behavioural test more than once. In a meta-analysis, the mean repeatability value was 0.37 (reviewed by Bell et al. 2009). In general, the term repeatability is used when assessing the accuracy of specific measurements (Nakagawa and Schielzeth 2010). In animal personality research, repeatability specifies the proportion of between-individual variance relative to the total phenotypic variance in a population for repeated measures of the same behaviour (Dingemanse et al. 2010).

The repeatability of a specific behaviour is usually calculated based on repeated observations in the same context and life history phase. Repeatability estimates from observations across contexts or life history phases have also been reported as “repeatability”, but the alternative terms, cross-context repeatability (e.g. Laskowski and Bell 2013) or consistency (e.g. Wuerz and Krüger 2015), allow us to better specify the different information content of these estimates. The proportion of between-individual variance among measurements from different contexts or life history phases characterizes the stability of individual behaviour across a longer time interval or across changes in the environment. We here restrict the use of the term *repeatability* to estimates of the proportion of between-individual variance from observations within the same life history phase. We define *consistency* of behavioural traits as the proportion of between-individual variance from observations at different ontogenetic stages. We use the term personality trait for behavioural traits that are repeatable and consistent and can hence contribute to animal personality. A behavioural type is characterized by a particular combination of trait expressions in two or more personality traits (Bell 2007).

There is growing evidence that behavioural syndromes can be less stable across lifetimes than previously assumed (Wuerz and Krüger 2015; Fischer et al. 2016; but see Gyuris et al. 2012). Developmental changes could reveal that underlying mechanisms of animal personalities (Stamps and Groothuis 2010b; Trillmich and Hudson 2011) as different experiences during development can shape behavioural types. Also, the hormonal constitution undergoes considerable change during the phase of vertebrate maturation, and this is likely to influence individual behavioural consistency (Stamps and Groothuis 2010a). Bell et al. (2009) already suggested to analyse repeatable behaviours between different age classes within species to investigate the potential impact of age and individual (sexual) experience on the repeatability and consistency of behavioural traits. Although their meta-analysis could not detect any differences in the repeatability of behaviours between juveniles and adults (Bell et al. 2009), a recent review by Brommer and Class (2015) reported evidence for lower behavioural repeatability in juveniles. Changes in behavioural repeatability and consistency across the life history do not contradict the existence of animal personality. They rather highlight the necessity to investigate personality traits across different life history phases to fully understand when and how animal personality arises and how long it persists. Recently, experiments have in fact shown that animal personality (based on repeatable behaviours) can arise during ontogeny (Polverino et al. 2016) but that it can also undergo a senescent decline in the wild (Class and Brommer 2016). Thus, understanding how repeatability (within life history stages) and consistency (between life history stages) differ between different developmental stages within a species is an important first step towards the definition and investigation of animal personality.

Réale et al. (2007) proposed measuring animal personality based on five behavioural traits that confer non-overlapping information on an animal’s behaviour and have been shown to affect fitness-relevant traits in a number of species: boldness, activity, exploration, sociability and aggression. We here investigated three of these traits in Eurasian harvest mice, as we considered them of particular interest in this solitary species (see also below): boldness (the tendency of an individual to take risks), activity (the general activity level of an individual) and exploration (an individual’s reaction to a new situation, for instance, novel objects or novel environments; see also Réale et al. 2007). Exploration was a consistent trait over a significant part of an individual’s lifetime in great tits (*Parus major*; Dingemanse et al. 2002) and zebra finches (*Taeniopygia guttata*; David et al. 2012). But while boldness, exploration and activity were repeatable behavioural traits over short periods of time in small rodents (e.g. Koolhaas et al. 1999; Boon et al. 2007; Lantova et al. 2011; Petelle et al. 2013), only activity seemed to be consistent across life history stages in that group (e.g. Kanda et al. 2012; Herde and Eccard 2013; but see Guenther et al. 2014).

Behavioural repeatability within, but no consistency across life history, phases can arise from developmental changes (see above). However, a simulation study showed that life history trade-offs can promote the evolution of behavioural types that are specific to particular life history phases (Wolf et al. 2007). Empirical data provided evidence that the level of boldness can vary between individuals depending on expected future reproductive success: in grey mouse lemurs (*Microcebus murinus*), young males had low current but high expected future reproductive success, while the opposite was true in older males (Dammhahn 2012). Hence, the trade-off between the investment into current and future reproduction differed between age classes. Dammhahn (2012) found that young male mouse lemurs were shyer than older males. She hypothesized that selection favoured young mouse lemurs that exhibited less risky behaviour because of the expected future fitness payoff, while older mouse lemurs benefitted most from (also risky) investments into the current reproductive effort. A trade-off between reproductive states may thus maintain animal personality variation in this species (Dammhahn 2012).

The repeatability and consistency of behavioural traits can also differ between the sexes (Schuett et al. 2010). In the abovementioned grey mouse lemurs, males were, on average, bolder than females and boldness was more repeatable in males than in females (Dammhahn 2012). Schuett et al. (2010) suggested that females prefer males that express consistent behavioural traits as reliable signals of quality. If so, the higher repeatability of boldness in male mouse lemurs could have resulted from sexual selection through female choice (Dammhahn 2012). Sex-specific natural selection can also cause sex differences in behavioural consistency. Male adult field crickets (*Gryllus integer*) behaved more shyly than juvenile males, while females showed consistent boldness across life history stages (Hedrick and Kortet 2012). Due to courtship callings, male crickets face a higher predation risk after metamorphosis than females (Hedrick and Kortet 2012). The sex difference in behavioural consistency seemed to result from those differences in costs and benefits of risk-taking behaviour between young and adult individuals (Hedrick and Kortet 2012). Overall, males tended to show more repeatable behaviours than females in a meta-analysis including a variety of behaviours in many different animal species, but this difference in repeatability between males and females may be biased due to the generally low repeatability of mate preference behaviours in females (Bell et al. 2009).

Here, we investigated the repeatability and consistency of three behavioural traits and one cognitive trait in the Eurasian harvest mouse (*Micromys minutus*), in males and in females, as well as in various life history phases. We measured the standard personality traits activity, boldness and exploration, as we expected them to differ consistently between individuals in our study organism like in other rodent species. Being a small prey species, levels of activity, boldness and exploration are likely to affect the survival of harvest mice critically. These traits affect predator avoidance behaviour, and they may show different adaptive optima depending on the body condition of an individual and on its specific environment. Thus, consistent between-individual differences in these behaviours may be expected in harvest mice. We calculated the repeatability of these traits within juveniles and within adults to investigate if individuals already differ consistently before maturation or if consistent between-individual behavioural differences arise later. Given the general theoretical prediction of a relationship between animal personality and cognition (Sih and Del Giudice 2012), we also investigated the repeatability of a spatial cognition trait. Spatial cognition can be defined as how animals acquire, process, store and use spatial information from the environment (Shettleworth 2010). This type of information can have a direct impact on the individual expression of the personality traits activity and exploration.

As a first step towards the exploration of a potential link between personality and spatial cognition, we investigated consistent between-individual differences in spatial cognition in harvest mice. We assumed that spatial cognition is an important cognitive ability in this species, as harvest mice, in their natural habitat, use a dense three-dimensional grid of various tussock and reeds. Within this compact vegetation, each individual uses several sleeping and breeding nests spread across different heights, and these nests are rebuilt repeatedly at new locations. Harvest mice thus have to acquire and store precise information about the location of their nests, and this information has to be updated frequently. The size of the hippocampus, a brain region involved in processing spatial information about the environment (Yaskin 2011), suggests indeed that the brain structure of harvest mice is specifically adapted to spatial orientation: it occupies 16.2% of the telencephalon, this is 4.6% more than in laboratory house mice (*Mus musculus*, 11.6% hippocampal volume of the telencephalon; West 1990).

We chose to measure object-based spatial recognition of a novel arm in a Y Maze and investigated the repeatability of this cognitive trait. Furthermore, we analysed the consistency of all studied traits across life history stages (before and after maturation, as well as before and after the first sexual contact). We considered it likely that developmental processes (e.g. hormonal changes during maturation), or in a natural environment expected changes (e.g. population density), differentially affect behaviours of harvest mice at different life history stages and may thus affect the consistency of individual behaviour.

Since little is known about the behaviour of harvest mice in a natural environment, and about their social and mating system, we based our predictions partly on what is known on personality traits in other small rodents. We expected to find repeatable behaviour within life history stages in juveniles, as well as in adult Eurasian harvest mice. Since activity was the most consistent behaviour in other rodents, we expected activity to be consistent across life history stages in our study species. We hypothesized that the expected future reproductive success differs between individuals that have not yet had sexual contact, and those that already experienced sexual contact, given the very short lifespan of harvest mice under natural conditions. Thus, risky behaviour may be less adaptive before the first mating than after the first mating, and we expected that boldness would not be consistent across life history stages. Dispersal patterns, which are probably influenced by an individual’s activity and exploration, are not well understood in harvest mice, but we presumed differences in the dispersal probability at different life history stages. Thus, we expected that activity and exploration would not be consistent as was also shown in previous studies of rodent personality. However, we assumed consistency in spatial recognition, as the motivation for and the reliance on spatial orientation are likely to be stable over lifetime. This is, because constantly returning to the sleeping nest or to the foraging habitat, for example, is likely to be adaptive within all life history stages. We further predicted that juvenile harvest mice are shyer than adult harvest mice due to high predation risk for all ages, but higher expected future reproductive success in young harvest mice. Little is known about mate choice in harvest mice and we can only explore sex differences in the behavioural consistency here. However, if females are the choosy sex, and if they prefer males that express consistent behavioural traits as reliable signals of quality (Schuett et al. 2010), we would expected less repeatable behaviour in female adult mice compared to males. We expected no repeatability differences between the sexes in juveniles.

We chose two approaches to estimate repeatability and consistency of behaviours: one approach included an additional random factor for the litter identity; the other approach was without this factor. Comparing repeatabilities obtained from both models allowed us to obtain a first indication on whether genetic or maternal effects contribute to behavioural repeatability. We had no predictions for this comparison, as there is no information so far on genetic or maternal effects on the repeatability of behaviour in Eurasian harvest mice. However, if we observe reduced estimates of repeatability with a litter effect in the model, compared to a model without that effect, this would mean that the observed repeatability is (partly) due to either genetic effects, direct maternal effects or other environmental effects that are common to litter mates.

## Methods

### Study species

The Eurasian harvest mouse is one of the smallest rodents in Europe with an average body mass of 7 g (Piechocki 2001). It is widely distributed in Europe and Asia (Krystufek and Kovacic 1984; Spitzenberger 1986) where it inhabits reed belts, high grass vegetation of wetland areas, grain fields and ruderal areas (Feldmann 1984; Spitzenberger 1986; Feldmann 1997). Within the Muridae, *M. minutus* is quite special as it is an excellent climber and lives mainly above the ground, where it feeds on seeds and insects (Piechocki 2001; Okutsu et al. 2012). The small body size and a long tail that can firmly cling to the vegetation are morphological adaptations to its preferred habitat (Frank 1957). Harvest mice live in unstable environmental conditions caused by seasonal changes of abiotic and biotic factors: rain, wind and drought affect the grassy habitat structure and food availability, and as in other rodent species, population sizes increase during the summer and show high peaks in autumn, followed by a strong population decline over winter (Piechocki 2001). Males and females build several spherical sleeping nests, mainly aboveground in dense vegetation (Piechocki 2001). These nests provide shelter from terrestrial predators and from water, as many habitats are at least periodically flooded. The mating system is not known, but only females build and use breeding nests and raise the young there on their own. The age of sexual maturation depends on environmental conditions (Frank 1957) but has been recorded between the ages of 40 to 50 days (Kubik 1952; Braun and Dieterlen 2005). There are no reliable data on the life span of harvest mice in the wild. Estimates for the average life expectancy range from two to 18 months (Kubik 1952; Piechocki 2001). While harvest mice can live up to 25 months in captivity (Schuster, personal observation), the maximum life span observed in natural or semi-natural populations was 11 and 14 months, respectively (Padilla 1999 and own observation).

In total, we tested 41 male and 42 female harvest mice from 34 litters (one to six offspring per litter). All animals stemmed from our laboratory population whose founding individuals (*N* = 26) originated from four different zoo populations. The mean inbreeding coefficient of the observed individuals was 0.15 (range 0–0.31). Mice were housed in polycarbonate cages of 60 cm length, 40 cm width and 58 cm height. Individuals were either kept separately or in pairs of equal sex with water and food (hay, grain seeds, fresh fruit and vegetables) ad libitum. The back wall of the cage was covered by a coco coir mat for climbing. For environmental enrichment, cages were additionally equipped with an artificial nest, a running wheel, a paper tube, a wooden branch and a sheaf of wheat, oat and spelt. All mice were kept at constant temperature (22 °C, range 21.0–23.5 °C) and light-dark cycle (LD 12:12 h). Animal husbandry and behavioural tests (see below) were permitted by the Regierungspräsidium Tübingen—Referat 35, reference number ZO 2/11.

### Experimental setup

We tested harvest mice in four different age classes (see Table 1) in three different behavioural tests. The number of tested animals differed between tests due to some exclusions (see also behavioural test descriptions). In total, 83 animals were tested at the age of 6 weeks (mean age = 44.5 days). Thirty-nine of these animals were tested at the age of 7 weeks (mean age = 51.7 days). We assumed that in these two age classes, animals were not fully mature yet, but developmental and hormonal changes may have already occurred due to starting maturation. For the third age class, we tested 52 of the initial 83 mice at the age of 12 weeks (mean age = 86.2 days). At this point, all animals were assumed to be mature, but none of them have experienced sexual contact yet. Finally, 47 individuals were tested at the age of 24 weeks (mean age = 174.9 days). Some of these (*N* = 16) were allowed to have sexual contact between week 12 and week 24. We measured the repeatability of behaviours in juvenile harvest mice before maturation using the data obtained in weeks 6 and 7 (subset repeatability; Table 1) and the repeatability of behaviours in adult mice after maturation using the data from weeks 12 and 24. Hereby, we analysed individuals which had no sexual experience (subset repeatability; Table 1). To test if sexual experience influences the consistency of the behavioural traits in adult harvest mice, we analysed the consistency of adult individuals (subset consistency, week 12 vs. week 24; see Table 1), which had sexual experience between tests. During the sexual experience phase, one female and one male spent 2 weeks together in a home cage giving them the opportunity to mate before being separated again. To measure consistency across different life stages (before and after maturation), we compared the age classes week 6 and week 12 (subset consistency; Table 1). Additionally, we compared mean levels of behaviours between males and females in young harvest mice (6 weeks) and in adults (12 weeks). Furthermore, we compared mean levels of behavioural traits between juvenile and adult harvest mice (week 6 vs. week 12).

**Table 1:**
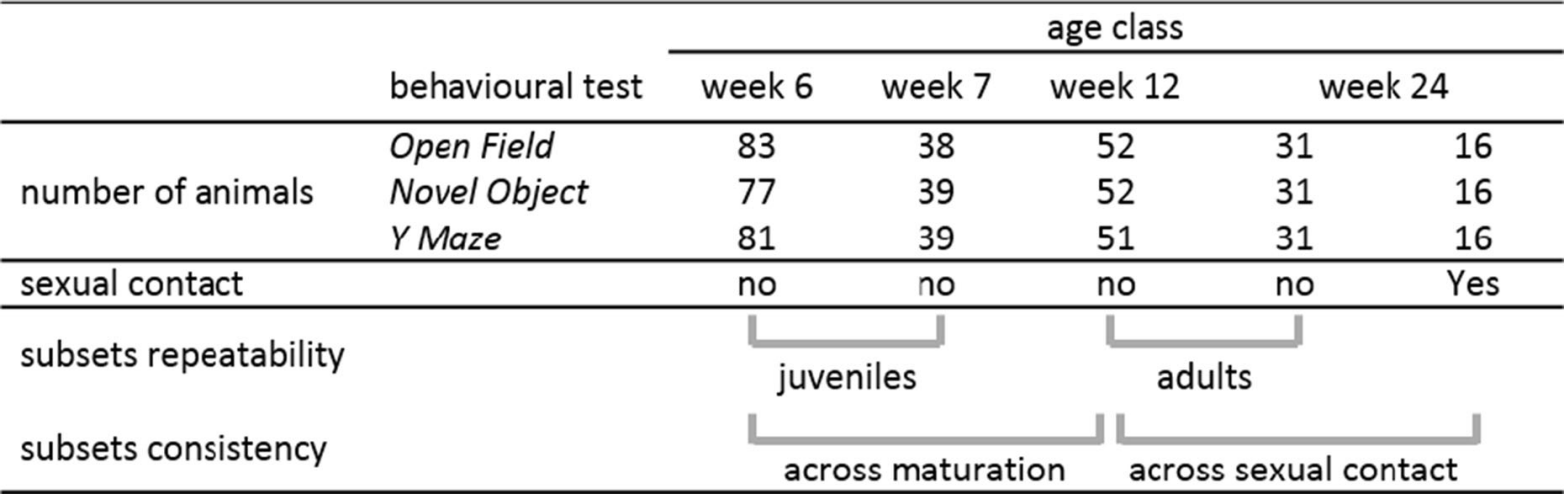
The number of tested animals in each of the three behavioural tests for the four different age classes (weeks 6, 7, 12 and 24)

### Behavioural tests

#### Open Field

We used a modified Open Field (OF) test (Archer 1973) to measure activity and boldness. In the OF test, rodents are expected to spend more time next to the wall of the arena due to their predisposition to avoid open space and the risk of avian predation (Archer 1973). The OF test is hence supposed to be a suitable setup to measure boldness in rodents (Herde and Eccard 2013). We used a standard mortar bucket (diameter 51 cm), which we painted with white colour for efficient tracking results. Using automated video tracking (EthoVisionXT, Version 5, Noldus), we measured the total distance moved (in cm) during 4.5 min and the time (in seconds) spent at the inner part of the arena (unsafe area, diameter 31 cm) as parameters for activity and boldness, respectively. Mice were released in the middle of a round arena. Tracking started when the animal reached the outer zone of the arena next to the wall for the first time (safe area, 10 cm wide). We excluded one record from the analysis (see Table 1; week 7), because one mouse did not move during the OF experiment and spent the whole time in the middle of the arena.

#### Novel Object

To quantify exploratory behaviour, we used a Novel Object (NO) test (Chitty and Shorten 1946). This was conducted directly after the OF test to reduce further stress as animals were already habituated to the arena setting. We used different novel objects for each trial: a small plant pot, a plastic box and a glass bowl. Each object was about the same size of the mice, and animals were able to explore it from all sides and also from the top by climbing on it. We recorded exploratory behaviour manually for 5 min after the first contact with the novel object as the time (in seconds) the animal spent in physical contact with the novel object (touching the object with its head, sniffing at the object from a maximal distance of 1 cm or climbing onto the object). Accidental contacts, e.g. with the tail, were not quantified as exploration. Since this test was initiated only after all other tests had been established, only 77 animals ran through the NO test at the age of 6 weeks (see Table 1).

#### Y Maze

We quantified spatial recognition in an adapted Y Maze (YM) arena (Montgomery 1955). This test relies on the assumption that rodents explore novel environments more than already known environments (Hughes 1968). Thereby, known environments can be recognized through object cues. In our setup, spatial recognition was only based on objects placed around the arena. The YM was made of transparent plastic; each of the three arms was 29 cm long, 3.5 cm wide and 10 cm high. We used a set of extra maze cues, which the animals knew from their home cages: small plant pots, stands of a running wheel, paper tubes, wooden sticks, clothes pegs, small glass bowls, pieces of coco coir mat and stems of straw. Each arm of the YM was surrounded by plain cardboard such that the animal could only see the object cues placed around the arm it was in. Visual cues were randomized between arms and animals, as was the position of the start arm. The YM test consisted of two trials. During the first trial, one of the arms was locked (unknown arm) and the animal could explore only two arms (start and known arm) for 15 min. After 1 h, the mouse was released again into the YM at the same start arm. In this second trial, all three arms were accessible. In the cognition literature, the behaviour of spending more time in the unknown arm of a Y Maze in the second trial is commonly defined as spatial recognition in the sense that an animal recognized that there is an unknown environment (e.g. Dellu et al. 1992). We video tracked animals using EthoVisionXT software and measured the total distance moved (in centimetres) during the first 5 min of the second trial as parameter for activity. The time (in seconds) spent in the unknown arm during the first 5 min of the second trial was recorded as parameter for spatial recognition. Two mice sat motionless in the start arm during the entire trial, and one animal ran straight to the end of the known arm and sat there motionless during the entire trial. These observations confer no information about whether the animal recognized the new arm and were deleted from the YM dataset (see Table 1).

### Data analysis

#### Test for confounding factors using linear mixed models

We performed all statistical analyses using R software, version 3.0.1 (R Core Team 2013). The dependent variables were the measured behaviours: distance moved in OF and YM (activity), time spent in unsafe area of the OF (boldness), time exploring NO (exploration) and time spent in unknown arm of the YM (spatial recognition). We tested for confounding factors of the experimental setup and of individual characteristics on the measured behaviours by fitting linear mixed models (LMMs) using the “nlme” package (Pinheiro and Bates 1996) in R. For each behavioural trait, we included the following confounding factors into the full model: sex, trial number (the number of test trials of each mouse) and housing condition (whether the mouse was kept individually or in a group of two in the home cage) as fixed factors and body mass, test date and test time as fixed covariates. The individual ID was included as a random factor. As males and females might differ in their activity rhythms, we included the interaction sex × test time. Further, female harvest mice are larger than males (Piechocki 2001), and this dimorphism might influence individual behaviours of sexes differently. We therefore also included the interaction sex × body mass in the full model. As the NO test was recorded manually by one of two observers, we also included the ID of the observer to linear models fitting exploratory behaviour. It was not possible to record data blind because our study involved focal animal observations. We applied backward stepwise reduction of the full models by excluding non-significant interactions first, followed by non-significant fixed effects if *p* > 0.05. The random factor of the individual ID remained in all models to account for repeated measures of individuals. All deleted confounding factors were added to the final (reduced) model again one by one to avoid missing a significant effect due to the order in which factors were deleted from the model. However, none of the deleted confounding factors had a significant effect if added again to the final model. We conducted this analysis separately for the four subsets of the data, as those subsets were then used to calculate repeatability or consistency (see the “Experimental setup” section; Table 1). All dependent variables and all covariates were centred and scaled to ensure model convergence. We checked all models for normal distribution of model residuals. We present final models with the remaining significant effects, which we retained for subsequent repeatability analyses. As we were interested in behaviour differences between males and females, and between juvenile and adult mice, we compared mean behaviours between those groups. The respective effect sizes from the confounding factors sex and trial number provided information on significant differences. We present group means ± standard errors of behavioural traits in the text (untransformed values).

#### Estimations of repeatability and consistency using linear mixed models

We applied LMMs (following Nakagawa and Schielzeth 2010) to estimate the repeatability and consistency of the behaviours. We thereby chose two approaches: one approach included an additional random factor for the litter identity; the other approach was without this factor. Personality may or may not have a genetic basis but is probably always also (and in some cases entirely) shaped by environmental influences (e.g. Nicolaus et al. 2016). Theory (e.g. Stamps and Groothuis 2010a, b; Trillmich and Hudson 2011) and recent experimental evidence (Polverino et al. 2016) show that personality can arise during ontogeny. Therefore, genetic effects, maternal effects, common environmental effects and interactions between siblings all play a contributing role in shaping personality in adult animals. Repeatable behaviour is a basic prerequisite for animal personality, and it can also arise from both, genetic and environmental effects on behaviour expression. This means that animals can behave repeatedly because their alleles affect behaviour to a significant extent or because their past experience within their environment causes a certain behavioural expression. The comparison between models accounting for a litter effect and models without that litter effect allowed us to obtain a first indication on whether genetic or maternal effects contribute to behavioural repeatability.

The repeatability of behavioural traits was analysed using the package “rptR” (Nakagawa and Schielzeth 2010) in R. We estimated *adjusted repeatability* (an estimate that adjusts for confounding effects; see Nakagawa and Schielzeth 2010) by including confounding effects that we identified from LMMs before (see above). We used untransformed dependent variables for these estimations. We estimated LMM-based repeatability applying non-parametric bootstrapping. We display asymptotic 95% confidence intervals (CIs) for parameter estimates based on 1000 bootstrapping runs and 1000 permutations. When using LMM-based repeatability estimation, the package rptR uses algorithms that do not converge if *R* is close to zero or negative. In this case, CIs and *p* values are shown as NA, and we concluded that *R* is zero. We used Bonferroni correction to account for multiple comparisons. Our significance threshold was then *p* ≤ 0.013, as we calculated four different repeatability values per behavioural trait (two age classes times two estimation methods) and four different consistency measurements per behavioural trait (two experimental groups times two estimation methods). The package *rptR* applies parametric bootstrapping for confidence interval estimation, but randomization for inference testing (*p* values). These two different methods may lead to non-congruent conclusions. In some of our analyses, *p* values indicated significance, although the confidence interval included zero. We here interpret effect sizes in combination with the confidence intervals and conclude that very small estimates of repeatability in combination with a confidence interval that includes zero do not suggest repeatability, even if the *p* value suggests significance. We consider repeatabilities larger than 0.1 as weak evidence, even if the estimated confidence interval includes zero. We display results of two different LMMs: LMM-based repeatability with litter ID as a random factor and models without litter ID. As tested individuals originated from 34 different litters (one to six offspring per litter), this allowed us to investigate whether differences between litters due to genetic variation and/or direct maternal effects contributed to the repeatability estimation of the tested behaviours. We compared two estimates of repeatability from different LMMs applying the overlapping confidence intervals method as suggested by Payton et al. (2003). Depending on the ratio of the standard errors of the two repeatability estimates, we calculated 84–87% CIs for these estimates. We considered two estimates of repeatability significantly different, if their CIs did not overlap. For details, see Payton et al. (2003). All CIs are given in squared brackets.

## Results

### Effects of confounding factors

Univariate LMMs revealed some significant fixed effects (Table 2) which we then included in the repeatability and consistency estimations (see below). The trial number had an effect on activity and on boldness in juvenile harvest mice (Table 2). In the second trial (week 7), juveniles were less active in the OF and in the YM and they were bolder in the OF than in the first trial (week 6). There was an effect of the observer identity on juvenile behaviour in the NO test, and the housing condition affected the behaviour of adult mice in that test: mice kept in pairs were more explorative than individually housed animals (see Table 2). We found no significant confounding effects on spatial recognition.

**Table 2:** Results of final LMMs with confounding effects on personality traits and spatial recognition

	Trait	Significant effect	Estimate ± SE	*p*
Juveniles (week 6 vs. 7)	Activity OF	Trial number	−0.30 ± 0.15	0.049
		Test date	+0.34 ± 0.11	0.005
	Boldness	Trial number	+0.53 ± 0.15	<0.001
	Activity YM	Trial number	−0.46 ± 0.18	0.017
	Exploration	Observer	+0.51 ± 0.20	0.016
	Spatial recognition	None		
Adults (week 12 vs. 24)	Activity OF	Housing condition	+0.99 ± 0.22	<0.001
		Sex	−0.55 ± 0.22	0.021
		Trial number	−0.47 ± 0.15	0.005
	Boldness	Sex	+0.85 ± 0.21	<0.001
	Activity YM	None		
	Exploration	None		
	Spatial recognition	None		
Before and after maturation (week 6 vs. 12)	Activity OF	Test Date	+0.33 ± 0.12	0.008
		Sex	−0.38 ± 0.18	0.042
		Trial number	−0.27 ± 0.12	0.025
	Boldness	Trial number	+0.76 ± 0.13	<0.001
	Activity YM	Trial number	−0.31 ± 0.14	0.032
	Exploration	None		
	Spatial recognition	None		
Before and after sexual contact (week 12 vs. 24)	Activity OF	None		
	Boldness	Sex	+0.69 ± 0.25	0.01
		Trial number	−0.50 ± 0.21	0.03
	Activity YM	None		
	Exploration	None		
	Spatial recognition	None		

All full models included the individual ID as random factor; sex, trial number and housing condition as fixed factors; body mass, test date and test time as fixed covariates, as well as the interactions sex × test time and sex × body mass

*SE* standard error, OF ﻿Open Field﻿ test, YM Y Maze test

### Effects of sex and age on the mean expression of behaviours

In juvenile harvest mice, males and females did not differ in their behaviour. Among adult harvest mice, females were bolder (*x̅* = 77.3 ± 6.0 s) and less active in the OF (*x̅* = 3506.9 ± 295.5 cm) than males (boldness *x̅* = 45.6 ± 4.4 s; activity *x̅* = 4374.5 ± 369.2 cm). This sex difference in boldness was also significant in adult mice tested before and after their first sexual contact (females *x̅* = 64.6 ± 7.0 s; males *x̅* = 42.2 ± 5.2 s). Furthermore, mice were significantly bolder before their first sexual contact than after (*x̅* = 56.0 ± 5.2 and 42.2 ± 8.6 s, respectively). We also found that the level of boldness was lower in juveniles that were tested before maturation (week 6) than in adult mice tested after maturation (week 12; *x̅* = 30.4 ± 2.8 and 56.0 ± 5.2 s, respectively). However, juveniles were more active in the OF and YM than adults (OF juveniles *x̅* = 4115.5 ± 247.6 cm and OF adults *x̅* = 4172.6 ± 318.1 cm; YM juveniles *x̅* = 1765.8 ± 91.4 cm and YM adults *x̅* = 1540.1 ± 143.2 cm). We did not find any differences between juveniles and adults in their exploratory behaviour or in spatial recognition.

### Repeatability

LMMs without the litter effect revealed that Eurasian harvest mice showed repeatable behaviours in both age classes (Table 3; LMM without litter effect). Activity in the OF test was highly repeatable in juveniles and adults (*R* = 0.54 ± 0.10 and *R* = 0.49 ± 0.13, respectively). Activity in the YM was repeatable in both age classes, but adult behaviour was more repeatable than juvenile behaviour, shown by non-overlapping 85% CIs (*R* = 0.69 ± 0.09 [0.56, 0.80] and *R* = 0.16 ± 0.14 [0, 0.40], respectively). Boldness was also repeatable in juvenile and adult harvest mice (*R* = 0.57 ± 0.11 and *R* = 0.20 ± 0.16, respectively). The higher *R* value for juvenile mice did not differ significantly from the lower value for adults (85% CIs [0.41, 0.70] and [0, 0.44], respectively). Similarly, exploration seemed more repeatable in juvenile mice than in adults, but the difference was not significant (84% CIs *R* = 0.40 ± 0.12 [0.26, 0.59] and *R* = 0.18 ± 0.15 [0, 0.42], respectively). Recognition of the novel arm in the YM was not repeatable in juveniles (*R* = 0.00), but adult mice behaved repeatably in this test (LMM without litter effect *R* = 0.20 ± 0.15, *p* = 0.004). In juveniles, the activity in the YM was only repeatable in males, not in females (*R* = 0.64 ± 0.13 and *R* = 0.00, respectively; data not shown). Secondly, in adult harvest mice, boldness was only repeatable in females, not in males (*R* = 0.58 ± 0.19 and *R* = 0.00, respectively; data not shown). Other repeatability estimates did not differ between the sexes.

**Table 3:** Results of repeatability analyses showing *R* values calculated for each personality trait and for spatial recognition per experimental group (juveniles and adults)

Experimental group	Trait	Fixed effects in LMM	LMM without litter effect				LMM with random litter effect		
			*N*	*R* ± SE	95% CI	*p*	*R* ± SE	95% CI	*p*
Juveniles (week 6 vs. 7)	Activity OF	Trial number + test date	83/38	0.54 ± 0.10	[0.35, 0.73]	*<0.001*	0.45 ± 0.13	[0.20, 0.70]	*<0.001*
	Boldness	Trial number	83/38	0.57 ± 0.11	[0.32, 0.73]	*<0.001*	0.27 ± 0.13	[0, 0.53]	0.038
	Activity YM	Trial number	81/39	0.16 ± 0.14	[0, 0.47]	*<0.001*	0.05 ± 0.11	[0, 0.37]	0.359
	Exploration	Observer	77/39	0.40 ± 0.12	[0.19, 0.66]	*<0.001*	0.39 ± 0.14	[0.17, 0.56]	0.004
	Spatial recognition		81/39	0.00	NA	NA	0.00	NA	NA
Adults (week 12 vs. 24)	Activity OF	Trial number + sex + housing condition	52/31	0.49 ± 0.13	[0.21, 0.72]	*<0.001*	0.49 ± 0.17	[0.01, 0.69]	*0.006*
	Boldness	Sex	52/31	0.20 ± 0.16	[0, 0.53]	*<0.001*	0.20 ± 0.15	[0, 0.49]	0.161
	Activity YM		51/31	0.69 ± 0.09	[0.49, 0.82]	*<0.001*	0.11 ± 0.10	[0, 0.34]	0.101
	Exploration		52/31	0.18 ± 0.15	[0, 0.49]	*0.006*	0.00 ± 0.11	[0, 0.36]	1.000
	Spatial recognition		51/31	0.20 ± 0.15	[0, 0.53]	*0.004*	0.00	NA	NA

Sample sizes are given: first trial/ second trial. Italic *p* values were significant after Bonferroni correction (*p* < 0.013). NA indicates that the model did not converge because of too small/negative value of *R*

*﻿OF* Open Field test, *YM* Y Maze test

### Consistency

LMMs without the litter effect showed that Eurasian harvest mice behaved consistently before and after maturation, as well as before and after their first sexual contact (Table 4; LMM without litter effect). Harvest mice tested before (week 6) and after (week 12) maturation were consistently active, bold and explorative (all *R* > 0.39; see Table 4). Only the recognition of the novel arm in the YM was not consistent after Bonferroni correction (*R* = 0.11 ± 0.11, *p* = 0.010). Adult harvest mice also behaved highly consistently before (week 12) and after (week 24) their first sexual experience (Table 4; LMM without litter effect). Activity in the OF and YM, as well as boldness, were highly consistent (all *R* > 0.53). Exploration was also significantly consistent between the tests, although this *R* value was smaller (*R* = 0.18 ± 0.19). The only clearly inconsistent behaviour shown before and after first sexual contact was the recognition of the novel arm in the YM (*R* = 0.00 ± 0.15). None of the consistency estimates differed between the sexes (data not shown).

**Table 4:** Results of consistency analyses showing *R* values calculated for each personality trait and for spatial recognition per experimental group (before and after maturation, before and after sexual contact)

Experimental group	Trait	Fixed effects in LMM	LMM without litter effect				LMM with random litter effect		
			*N*	*R* ± SE	95% CI	*p*	*R* ± SE	95% CI	*p*
Before and after maturation (week 6 vs. 12)	Activity OF	Trial number + sex + test date	83/52	0.64 ± 0.07	[0.49, 0.77]	*<0.001*	0.47 ± 0.11	[0.27, 0.70]	*<0.001*
	Boldness	Trial number	83/52	0.39 ± 0.11	[0.17, 0.59]	*<0.001*	0.36 ± 0.13	[0.10, 0.58]	*0.002*
	Activity YM	Trial number	81/51	0.42 ± 0.11	[0.22, 0.63]	*<0.001*	0.07 ± 0.09	[0, 0.31]	0.237
	Exploration		77/52	0.41 ± 0.12	[0.13, 0.59]	*<0.001*	0.13 ± 0.11	[0, 0.39]	0.180
	Spatial recognition		81/51	0.11 ± 0.11	[0, 0.36]	*0.010*	0.00	NA	NA
Before and after sexual contact (week 12 vs. 24)	Activity OF		52/16	0.84 ± 0.06	[0.70, 0.92]	*<0.001*	0.77 ± 0.12	[0.46, 0.92]	*<0.001*
	Boldness	Sex + trial number	52/16	0.53 ± 0.15	[0.24, 0.82]	*<0.001*	0.53 ± 0.18	[0.04, 0.78]	*0.008*
	Activity YM		51/16	0.70 ± 0.15	[0.28, 0.87]	*<0.001*	0.00 ± 0.06	[0, 0.19]	0.500
	Exploration		52/16	0.18 ± 0.19	[0, 0.60]	*0.006*	0.00 ± 0.11	[0, 0.44]	0.500
	Spatial recognition		51/16	0.00 ± 0.15	[0, 0.51]	*0.009*	0.00	NA	NA

Sample sizes are given: first trial/ second trial. Italic *p* values were significant after Bonferroni correction (*p* < 0.013). NA indicates that the model did not converge because of too small/negative value of *R*

*OF* Open Field test, *YM* Y Maze test

### Results of linear mixed models with litter effect

When we included a random effect for the litter, we observed, in most cases, reduced values for repeatability and consistency (see Tables 3 and 4). The most pronounced reduction in *R* occurred in the models for activity in the YM. For this trait, the repeatability estimate in adults and both consistency estimates differed between the LMMs without and with the litter effect (repeatability in adults, 84% CIs [0.57, 0.80] and [0, 0.27]; consistency before and after maturation, 84% CIs [0.26, 0.59] and [0, 0.23]; consistency before and after sexual contact, 87% CIs [0.40, 0.84] and [0, 0.14], respectively). Other 84–86% CIs did not differ significantly between LMMs. However, with litter as random effect (and thus partly excluding potential genetic effects and non-genetic maternal effects from the estimation of *R*), activity in the OF was repeatable and consistent, boldness was consistent but its repeatability estimates did not reach significance and exploration was repeatable only in juvenile mice.

## Discussion

All tested behavioural traits were repeatable within life history stages and consistent across life history stages, with the only exception of spatial recognition. Activity, boldness and exploration hence fulfil the properties for an animal personality trait and could be components of a behavioural syndrome in Eurasian harvest mice, as it was also found in other rodents (Koolhaas et al. 1999; Boon et al. 2007; Boyer et al. 2010; Kanda et al. 2012; Herde and Eccard 2013). Spatial recognition was repeatable only in adult mice, not in juveniles. Further, we found less evidence that mice recognized the new arm of the Y Maze consistently before and after maturation, as well as before and after the first sexual contact.

### Effects of sex and age on the mean expression of behaviours

We found evidence that adult females were less active than males. This difference was significant in the OF test, but not in the YM test. In turn, adult males were on average shyer than females. We detected no sex differences in exploration or spatial recognition. There is little evidence for consistent sex differences in animal personality traits in rodents. In general, sex differences in behaviour of rodents are inconsistent and depend on species and behavioural trait (common voles, *Microtus arvalis*: Herde and Eccard 2013, but see Lantova et al. 2011; meadow voles, *Microtus pennsylvanicus*: Halliday et al. 2014; wild house mice, *Mus domesticus*: Auclair et al. 2013; laboratory house mice, *M. musculus*: Montiglio et al. 2010). Given our currently scarce knowledge on individual behaviour of our study species, conclusions on the selection pressures that may maintain sex differences in activity and boldness remain speculative.

Wolf et al. (2007) hypothesized that animals with high expected future reproductive success should be more risk averse than individuals with low expected future reproductive success. As a consequence, the individual state of an animal in the ongoing trade-off between current and future reproductive success is expected to affect the strength of selection for more risk-averse behaviour early in life and for more risky behaviour late in life. In our study, young harvest mice were indeed shyer than older mice, but juveniles were more active than older mice. Younger individuals have more to lose, as they did not yet have the opportunity to reproduce. A more cautious behaviour is likely to increase survival chances in the wild, where terrestrial and aerial predators pose a considerable risk to harvest mice. After maturation, selection may favour males that invest more in actively finding receptive females, and it may favour females that invest more in finding the best food sources and potential nest sites within their home range. It should be noted that juveniles behaved more boldly and were less active in the second trial than in the first (weeks 7 and 6, respectively). This suggests a habituation effect that may have contributed to the behaviour differences between juveniles and adults in our experiments. However, in the wild, habituation is very likely to contribute to behavioural changes across life history stages as well, as individuals get habituated to the specific environment in their home range and, as a benefit from that, can show more risky behaviour as soon as the exact location of food sources and hiding places are known. Thus, the here observed changes in boldness and activity across life stages could be the result of life stage specific selection on risk-averse behaviour. However, we also observed that harvest mice behaved more shyly after the first sexual contact, and this observation does not support our hypothesis.

### Repeatability

As expected, juvenile and adult harvest mouse behaviour was significantly repeatable (Table 3). Adult mice did not generally behave more repeatably than juveniles. Only activity in the YM, as well as spatial recognition (also measured in YM), was more repeatable in adults than in juveniles. However, our test for spatial recognition might not be adequate for juvenile harvest mice, as we observed that juveniles often climbed the walls of the YM trying to escape. Thus, this climbing activity contributed to both measurements taken in the YM: duration in the novel arm (spatial recognition) and distanced moved in total YM (activity). And this contribution may depend more on the motivation to climb the walls during the test, rather than intrinsic differences in activity and spatial recognition. This leads us to a cautious interpretation of the outcome of the spatial recognition test in juveniles. It might be preferable to design a test where mice cannot climb as much as in the YM. However, as activity in the OF test was highly repeatable in juveniles, we have no doubt that activity is a repeatable behaviour also in juvenile harvest mice.

Although two of the here presented values for repeatability were smaller than 0.2 (activity in the YM in juveniles and exploration in adults), our findings generally confirm the large body of evidence for the repeatable nature of these personality traits in many taxa (e.g. rodents: Koolhaas et al. 1999; Montiglio et al. 2012; Herde and Eccard 2013, birds: Dingemanse et al. 2002; David et al. 2012, reptiles: Carter et al. 2012; Bajer et al. 2015). Activity, boldness and exploration are particularly well studied animal personality traits, and there is growing evidence about potential changes in repeatability across life history stages (Brommer and Class 2015). Bajer et al. (2015) showed that juvenile European green lizards (*Lacerta viridis*) behaved significantly more repeatably than adults in risk-taking and exploratory behaviour. In our study, the repeatability of boldness and exploration were not influenced by age. However, there was an age effect on the repeatability of activity in the YM, but not so in the OF. This is in concordance with findings in zebra finches (*T. guttata*), where activity was repeatable in subadults but not in young and mature birds (Wuerz and Krüger 2015). We found some differences between the repeatability values of males and females. In adult harvest mice, boldness was only repeatable in females. This was in contrast to our expectation, as we assumed that males would behave more repeatably due to the sex role of the chosen sex. However, this result should be interpreted with care as we have to acknowledge the smaller sample sizes (N between 15 and 21 animals per group) compared to other data subsets, which may have influenced these results.

In sum, we found that Eurasian harvest mice already express repeatable behaviours as juveniles. Furthermore, all tested personality traits and a spatial cognition trait were repeatable in adults. Behavioural repeatability did not seem to change across life history stages, which suggest that behavioural types, if existent in this species, may be a stable phenomenon. As this is the first study on individual behaviours in this species, more investigations are warranted to fully understand which factors maintain the repeatability of behaviours here.

### Consistency

Eurasian harvest mice showed consistency in activity, boldness and exploration (Table 4; but note that the consistency estimate for exploration in adults was smaller than 0.2). This was in contrast to our expectations. We assumed that differences in the expected future reproductive success and in dispersal patterns at different life history stages would result in life history-specific behavioural patterns. However, although the mean behavioural expression changed (adult mice were more bold, but less active than juveniles; see above), the behavioural differences between individuals remained consistent across maturation and across the first sexual experience. During maturation and the first sexual contact, the relative magnitude of attained reproductive success and expected future reproductive success changes depending on individual experiences. The dynamics of this trade-off may result in a change of selection pressures on animal personality traits (Bell and Stamps 2004). However, these events did not seem to have an influence on the consistency of the studied personality traits in harvest mice. We tested adult mice at the ages of 3 and 6 months, which probably represent the entire mean life span of this species in the wild (Kubik 1952; Piechocki 2001). Hence, Eurasian harvest mice—at least under laboratory conditions—seem to behave consistently throughout their entire life span, and the short-term consistency of these behaviours was independent of maturational effects and sexual experience. Although, we acknowledge that the sample size to test for the influence of sexual experience was smaller compared to other subsets.

Bell and Stamps (2004) stated that individual behavioural consistency can vary across lifetime. In particular, they suggested a decline in consistency during sexual maturation, because correlations between behavioural traits may be restructured and become instable due to hormonal effects during this life history phase. Contradictory to our findings, other studies support this idea through data on inconsistent behaviour across individual maturation. In guinea pigs (*Cavia aperea*), Guenther et al. (2014) showed that the personality structure changed over different life history stages: while fearlessness and boldness were consistent over maturation, exploration was not, and correlations between behavioural traits changed during ontogeny. In common voles (*M. arvalis*; Herde and Eccard 2013) and Siberian dwarf hamsters (*Phodopus sungorus*; Kanda et al. 2012), activity was a consistent behavioural trait across different life history stages. Boldness, exploration, reactivity and orientation were not consistent. In contrast, activity, boldness and exploration were consistent in some studied birds and insects (Dingemanse et al. 2002; David et al. 2012; Fisher et al. 2015). A study on firebugs (*Pyrrhocoris apterus*) showed that the personality structure can also be consistent across life history stages: Gyuris et al. (2012) found that firebugs behaved consistently and showed consistent trait correlations across larval and adult stages, even past final ecdysis. Thus, even major changes between life history stages are not necessary connected to inconsistent individual behaviour. To date, no comprehensive theory has been developed to predict which factors would favour or disfavour consistent behaviour in specific species. This is in part due to the fact that consistent behaviours can arise from two sources: natural selection for stable behavioural types or physiological constraints that prevent flexible behaviour. The relative contribution of these to the expression of personality traits is currently largely unknown. It could be that in harvest mice, consistently behaving animals were favoured by selection. In a frequently changing environment, short-lived animals may save energy and avoid erroneous reactions if they restrain from fast behavioural changes in response to environmental cues. Adopting an individual behavioural tactic that suits best the individual constitution may be advantageous. Alternatively, or in addition, physiological constraints may maintain consistent behaviours across the short lifespan.

We also tested if harvest mice behaved repeatably and consistently when they explored a novel arm in a Y Maze. This setup measured an individual’s ability to recognize a new spatial environment based on object cues. Unlike in the personality traits discussed above, we observed less evidence for consistency in spatial recognition. While the performance in the Y Maze was repeatable among adult harvest mice, it was not consistent before and after the first sexual contact. Since there was no mean difference in the performance before and after sexual experience, but individuals did not behave consistently before and after sexual experience, we hypothesize that sexual experience affected the ability or the motivation of individual mice for this cognitive task. Spatial orientation and recognition can rely on the spatial arrangement of specific cues. We offered object cues in our test trials and excluded olfactory cues. In nature, harvest mice can certainly also use olfactory cues or environmental geometry for orientation. Individual harvest mice may also differ in which cues they prefer to rely on, and furthermore, specific experiences (like maturation or first sexual contact) may change the individual priority of used cues. Thus, if individual cue perception and cue use changes during the life of the mice (in our experiment: between the two test trials), this could have contributed to the observed inconsistency in spatial recognition in our test setup. However, it should be noted that the low sample size of adult mice tested after their first sexual experience (*N* = 16) only permits preliminary conclusions.

For a long time, the main interest in animal cognition research focussed on the comparative evaluation of cognitive abilities between species. Therefore, little is known about individual variation, repeatability and consistency of cognitive traits within species. We here provided a first evidence for the repeatability (in adults) and consistency (over maturation) of spatial recognition through object cues in harvest mice. Typical animal personality traits such as activity and exploration may correlate with particular cognitive styles (repeatable cognitive behaviour) to form a cognitive syndrome (Griffin et al. 2015; Sih and Del Giudice 2012). An assumption to this is repeatability and consistency of cognitive traits. Spatial recognition as tested in our setup meets this assumption and may thus be used for a test of correlations between personality and spatial cognition in harvest mice.

### Effect of the litter

Repeatability of a behavioural trait is the fraction of total trait variance that occurs between individuals, due to additive and non-additive genetic effects, the permanent environment effect, and other effects that cause differences between individuals, such as direct maternal effects (Falconer et al. 1996; Nakagawa and Schielzeth 2010). When we included the grouping factor litter as a random factor in the LMM analyses, the between-individual variance that is due to differences between litters was attributed to that random effect and could hence not contribute to the estimate of *R*. Accordingly, all models with the litter effect, except two (OF activity and boldness in adults), estimated lower *R* values than the corresponding models without the litter effect. While those *R* values present a biased estimate, as genetic and early environmental contributions to behavioural repeatability are partly excluded, they can give an indication on whether heritability or direct maternal effects contribute to the repeatability of a trait. Reduced estimates of repeatability (or consistency) with a litter effect in the model, compared to a model without that effect, indicate that the observed *R* value is (partly) due to either genetic effects, direct maternal effects or other environmental effects that are common to litter mates and thus cause differences between litters. In this study, we found particular strong evidence for those effects on activity in the Y Maze, as *R* values for this behaviour decreased significantly when the litter effect was included. Further investigations using the full pedigree of all tested animals are needed to quantify genetic and non-genetic maternal effects on activity in harvest mice.

## Conclusion

Our results show that activity, boldness and exploration were repeatable in juvenile and adult Eurasian harvest mice. Furthermore, these behaviours were expressed consistently independent of age, maturation and individual sexual experience. Our findings show that behaviours in harvest mice meet two essential assumptions in animal personality research—repeatable and consistent between-individual differences (Réale et al. 2007). Further, we tested spatial recognition based on object cues in a Y Maze. This trait was repeatable in adult mice, but not in juveniles, and it showed little evidence for consistency across life history phases. More investigations are needed to better understand the repeatability and consistency of cognitive traits as this would be a prerequisite for further studies on cognitive syndromes (Sih and Del Giudice 2012).
